# Differential responses of two plant species with different ecological niches to long-term nitrogen and phosphorus addition in temperate meadow steppe

**DOI:** 10.3389/fpls.2025.1693163

**Published:** 2025-10-27

**Authors:** Li Chen, Dan Zhao, Yunrui Yang, Yi Wei, Zhihui Wang, Hongyi Wang

**Affiliations:** ^1^ Heilongjiang Bayi Agricultural University, Daqing, China; ^2^ Key Laboratory of Low-Carbon Green Agriculture in Northeastern China, Ministry of Agriculture and Rural Affairs, Daqing, China

**Keywords:** plant functional traits, nitrogen addition, phosphorus addition, temperatemeadowsteppe, Ecological niche

## Abstract

**Introduction:**

Global nitrogen (N) and phosphorus (P) deposition are fundamentally altering soil nutrient availability and species interactions in grassland ecosystems. However, the long-term interplay between N and P in shaping ecological niche differentiation among co-existing plant species remains poorly understood.

**Methods:**

Leveraging a decade-long nutrient addition experiment in a temperate meadow steppe, this study investigated the response strategies of the dominant upper-canopy grass *Leymus chinensis* and the subdominant lower-canopy forb *Potentilla bifurca* by analyzing their phenotypic and nutrient traits.

**Results:**

We found that *P. bifurca* exhibited greater phenotypic plasticity than *L. chinensis*, a difference that was amplified under combined NP addition. Notably, the two nutrient regimes drove opposing niche dynamics: N addition alone exacerbated P limitation, reducing phenotypic trait differences between the species (niche distance decreased from 0.12 to 0.07) and leading to niche convergence. In contrast, combined NP addition alleviated P limitation, enhanced the plasticity of *P. bifurca*, and drove niche separation (distance increased to 0.16). Correlation and random forest analyses revealed that the aboveground biomass of *L. chinensis* was primarily associated with N-related traits, whereas that of *P. bifurca* was strongly associated with P-use efficiency traits.

**Conclusion:**

Our results demonstrate that long-term N and P addition regulate interspecific competition by modulating soil N/P ratio and driving divergent trait responses, thereby shaping the niche partitioning between co-existing species.

## 1 Introduction

Grasslands are an important component of terrestrial ecosystems, playing an irreplaceable role in climate regulation, biodiversity maintenance, and human social development (Gomez-Casanovas et al., 2021). Since the Industrial Revolution, global N deposition has continued to increase (Stevens et al., 2018; Liu et al., 2022). As a hotspot region for N deposition, China’s exogenous N input alleviates N limitation, and also triggers a chain reaction in biogeochemical cycles by altering the soil–plant interaction network. The alteration of the N cycle causes originally N-limited plants to enter a “N saturation” stage, and leads to other nutrients, such as phosphorus, becoming new growth-limiting factors (Yu et al., 2023). The increase in available N can disrupt the N–P balance of ecosystems, prompting plants to adjust nutrient uptake strategies, and through changes in phenotypic plasticity and resource allocation strategies, alter interspecific competition patterns, reconstruct community structure, and modify ecosystem functions (Elser et al., 2009; Xu et al., 2022).

Plant functional traits are generally divided into phenotypic and nutrient traits, referring to measurable individual characteristics that play important roles in plant survival, growth, and reproduction. These traits can independently or jointly reflect ecosystem processes in response to environmental changes from individual to community levels (Meng et al., 2007). Changes in plant phenotypic traits can reflect plant responses to soil nutrients and the external environment, while changes in plant nutrient traits can reflect nutrient uptake capacity, nutrient use efficiency, and nutrient limitations (Myers-Smith et al., 2019; Guo et al., 2022). Considerable research has been conducted on the responses of plant functional traits to N addition and their adaptive strategies. For example, when environmental available N increases, plants often optimize photosynthesis and resource use efficiency by altering leaf structural traits (such as leaf dry matter content, leaf area, and specific leaf area), thereby improving their environmental adaptability (Xu et al., 2023). In addition, in multiple grassland ecosystems, long-term and continuous N addition not only leads to increased leaf N content in fast-growing, high-resource-demand species, thereby improving leaf photosynthetic rate and biomass accumulation, but may also compress the survival space of other species, change plant community structure, and reduce community species diversity (Chen et al., 2016; Ma et al., 2025).

Compared with N, research on the effects of P on plant functional traits is relatively scarce. As an important element for plant growth, short-term P addition can significantly increase leaf P content and promote plant absorption and utilization of P, K, and other elements (Wang et al., 2024). However, the effects of long-term P addition are more complex: on the one hand, in ecosystems with severe P limitation, long-term P addition can continuously enhance plant P content and biomass, 69 enabling plants to adapt to environmental changes leveraging strategies such as reducing root-to- shoot ratio and increasing specific leaf area (Wang et al., 2021); on the other hand, excessive P may disrupt nutrient balance by altering the soil N:P ratio, thereby inhibiting plant N uptake. Over the long term, it can also lead to P accumulation in the soil pool, leading to P toxicity symptoms in plants (Liu et al., 2023). Some studies have found that N–P co-addition can produce an overyielding effect, and such synergistic effects may be more stable and pronounced in long-term experiments, helping maintain higher productivity and nutrient use efficiency (Zhang et al., 2022; Lu et al., 2025). However, the long-term effects of nutrient interactions on plant functional traits (such as nutrient uptake efficiency, ecological stoichiometry, and trait coordination effects) remain to be further studied.

Although considerable research has examined plant responses to N enrichment, studies on the long-term interactive effects of N and P addition, especially on the functional traits and niche dynamics of species occupying different canopy positions, remain limited. To address this knowledge gap, this study leverages a unique 10-year N and P addition experiment in a temperate meadow steppe, focusing on two key species with contrasting ecological niches: the dominant, tall grass *L. chinensis* and the subdominant forb *P. bifurca*. Specifically, we aim to address the following questions: (1) How do long-term N and P additions differentially affect the phenotypic plasticity and nutrient allocation strategies of *L. chinensis* and *P. bifurca*? (2) Do these changes in functional traits lead to niche convergence or divergence between the two species? (3) How are the observed plant responses linked to alterations in soil N/P stoichiometry? By answering these questions, we aim to reveal the mechanistic links between nutrient-induced soil changes, plant trait adaptation, and the reorganization of interspecific relationships, providing new insights into the ecological consequences of concurrent N and P deposition.

## 2 Materials and methods

### 2.1 Study Site

This study was conducted at the N and P addition control experimental platform of the Erguna Forest–Grassland Ecotone Research Station, Institute of Applied Ecology, Chinese Academy of Sciences (50°10′46″N, 119°22′56″E). The region has experienced a cold temperate climate, with an annual mean temperature of approximately −2.3 °C and an average annual precipitation of approximately 360 mm over the past 50 years. The terrain is relatively flat, the soil type is alkaline chernozem, and the grassland type is meadow steppe dominated by *L. chinensis*. The region experiences a background atmospheric N deposition of approximately 5.2-18.7 g N m^-2^ a^-1^, and the soil is historically deficient in available P (Yang et al., 2012; Wang et al., 2024), providing a relevant context for our nutrient addition treatments. The experimental site has been under enclosure management since 2013, and N and P addition treatments have been conducted since 2014.

### 2.2 Experimental design

This study employed a completely randomized block design with five blocks to account for spatial heterogeneity. Each block contained five plots (8 m×8 m), with a 1-m buffer strip between adjacent plots, resulting in a total of 25 experimental plots. The design focused on a gradient of P addition under a constant N background to precisely investigate the N-P interaction and identify potential thresholds of P effect. Five treatments were randomly assigned to the plots within each block: control (CK, no fertilizer), N addition only (10 g N m^-2^ a^-1^, designated as NP0), and combined N and P addition at three rates (10 g N m^-2^ a^-1^ plus 2, 4, or 8 g P m^-2^ a^-1^, designated as NP2, NP4, and NP8, respectively). Urea: CO(NH_2_)_2_ was used as the N source, and calcium superphosphate main component: Ca(H_2_PO_4_)_2_ was used as the P source. Fertilizers were applied once annually in late May by evenly broadcasting by hand before grass regreening.

### 2.3 Sample collection and measurement

In August 2023, during peak biomass, plant community surveys and functional trait measurements were conducted. We selected two key species that differ in canopy position and ecological strategy: the dominant, upper-canopy perennial grass *Leymus chinensis* (Trin.) Tzvel., and the subdominant, lower-canopy perennial forb *Potentilla bifurca* L. Five healthy, intact individuals of each species from each plot (n = 25 per species per treatment) were sampled for functional trait measurements. Stem diameter (SD) was measured using a digital vernier caliper (Mitutoyo, Kawasaki, Japan). Plant height (H) and stem length (SL) were measured with a steel ruler. Plants were clipped at ground level, sealed in envelopes, and oven-dried at 65 °C for 48 hours to constant weight to determine aboveground biomass (AGB). For leaf traits, five fully expanded, healthy leaves per individual were collected, placed in sealed bags with moist filter paper, and stored in a cool box for transport. In the laboratory, after rehydration to full turgidity, surface water was gently blotted. Leaf thickness (LT) was measured with a digital micrometer (DLX-TGM3124, Delexi Electric, China). Leaf area (LA) was determined by scanning leaves and analyzing the images with Image J software (National Institutes of Health, USA). Leaves were then oven-dried to constant weight to determine leaf dry weight (LDW), and specific leaf area (SLA) was calculated as LA/LDW. Dried plant samples were separated into stems and leaves to determine stem dry weight (SDW) and LDW. The dried samples were ground, digested with concentrated H_2_SO_4_ and H_2_O_2_, and then stem nitrogen content (SNC) and leaf nitrogen content (LNC) were determined using the Kjeldahl method. Stem phosphorus content (SPC) and leaf phosphorus content (LPC) were determined using the molybdenum–antimony colorimetric method.

Soil samples were collected concurrently from the 0–10 cm depth at five random points within each plot using a soil auger (7-cm diameter). Samples from the same plot were mixed homogenously, passed through a 2-mm sieve to remove visible roots and debris, air-dried, and stored for analysis. Soil available P (AP) was extracted with sodium bicarbonate and measured by molybdenum–antimony colorimetry. Soil ammonium N (NH_4_^+^-N) and nitrate N (NO_3_^-^-N) were extracted with 2 M KCl and determined by the indophenol blue colorimetric method and phenol disulfonic acid method, respectively. Soil total N (TN) was determined using the Kjeldahl method after digestion with H_2_SO_4_, and total P (TP) was determined by molybdenum–antimony colorimetry after NaOH fusion.

### 2.4 Data analysis

All data were organized and preliminarily processed using Microsoft Excel. Statistical analyses were performed using SPSS 22.0 (SPSS Inc., USA). One-way analysis of variance (ANOVA) followed by Duncan’s multiple range test (P< 0.05) was used to assess the significant differences among treatments. The following indices were calculated:


$$Inorganic\;Nitrogen(IN,mg/kg)=NH_{4}^{+}+NO_{3}^{-}$$

Proportion of Available Phosphorus(PAP)=PA/TP

Proportion of Inorganic Nitrogen(PIN)=IN/TP

$$Proportion\;of\;Organic\;Nitrogen\;(PON,mg/g)=TN-NH_{4}^{+}-NO_{3}^{-}$$

Phenotypic Plasticity Index (PPI)=(MAX−MIN)/MAX

Stem−to−Leaf Ratio(SLR)=SDW/LDW

$$Leaf\;Volume\;(LV,m^{2})=LA\times LT$$

Total Stem Carbon Mass (TSCM,mg)=SCC×SDW

Total Stem Nitrogen Mass (TSNM,mg)=SNC×SDW

Total Stem Phosphorus Mass (TSPN,mg)=SPC×SDW

Total Leaf Carbon Mass (TLCM,mg)=LCC×LDW

Total Leaf Nitrogen Mass (TLNM,mg)=LNC×LDW

Total Leaf Phosphorus Mass (TLPM,mg)=LPC×LDW

Total Plant Carbon Mass (TPCM,mg)=TSCM+TLCM

Total Plant Nitrogen Mass (TPNM,mg)=TSNM+TLNM

Total Plant Phosphorus Mass (TPPM,mg)=TSPM+TLPM .


Plant Carbon Content(PCC,mg/g)=TPCM/AGB

Plant Nitrogen Content(PNC,mg/g)=TPNM/AGB

Plant Phosphorus Content(PPC,mg/g)=TPPM/AGB

Plant Nitrogen to Phosphorus ratio (PN/P)=PNC/PCC

Plant Carbon to Phosphorus ratio (PC/P)=PCC/PPC

Plant Carbon to Nitrogen ratio (PC/N)=PCC/PNC

Plant Nitrogen Nutrient Efficiency Ratio (PNNER,g/mg)=1/PNC

Plant Phosphorus Nutrient Efficiency Ratio (PPNER,g/mg)=1/PPC

Leaf Nitrogen Nutrient Efficiency Ratio (LNNER,g/mg)=1/LNC

Leaf Phosphorus Nutrient Efficiency Ratio (LPNER,g/mg)=1/LPC

Stem Nitrogen Nutrient Efficiency Ratio (SNNER,g/mg)=1/SNC

Stem Phosphorus Nutrient Efficiency Ratio (SPNER,g/mg)=1/SPC

Stem−to−Leaf Total Carbon Mass Ratio (SLTCMR)=TSCM/TLCM

Stem−to−Leaf Total Nitrogen Mass Ratio (SLTNMR)=TSNM/TLNM

Stem−to−Leaf Total Phosphorus Mass Ratio (SLTPMR)=TSPM/TLPM

Stem−to−Leaf Total Carbon Content Ratio (SLTCCR)=SCC/LCC

Stem−to−Leaf Total Nitrogen Content Ratio (SLTNCR)=SNC/LNC

Stem−to−Leaf Total Phosphorus Content Ratio (SLTPCR)=SPC/LPC

Leaf Biomass Efficiency Ratio (LBER,g/g)=AGB/LDW

Productivity per Leaf Area (PLA,g/cm²)=AGB/LA

Multivariate analyses and advanced statistical modeling were conducted in R (version 4.3.1, R Foundation for Statistical Computing). Principal component analysis (PCA) of soil properties and principal coordinates analysis (PCoA) of plant traits were performed using the vegan package (v2.6-4) to visualize treatment effects. Spearman’s correlation analysis was used to construct trait correlation matrices. The random forest model, a machine learning algorithm for identifying important predictors, was built using the randomForest package (v4.7-1.1) to rank the importance of functional traits in explaining aboveground biomass (AGB), with the model run with 1000 trees. All graphs were generated using Origin 2024 (OriginLab Corporation, USA).

## 3 Results

### 3.1 Effects of N and P addition on soil physicochemical properties

**Figure 1:**
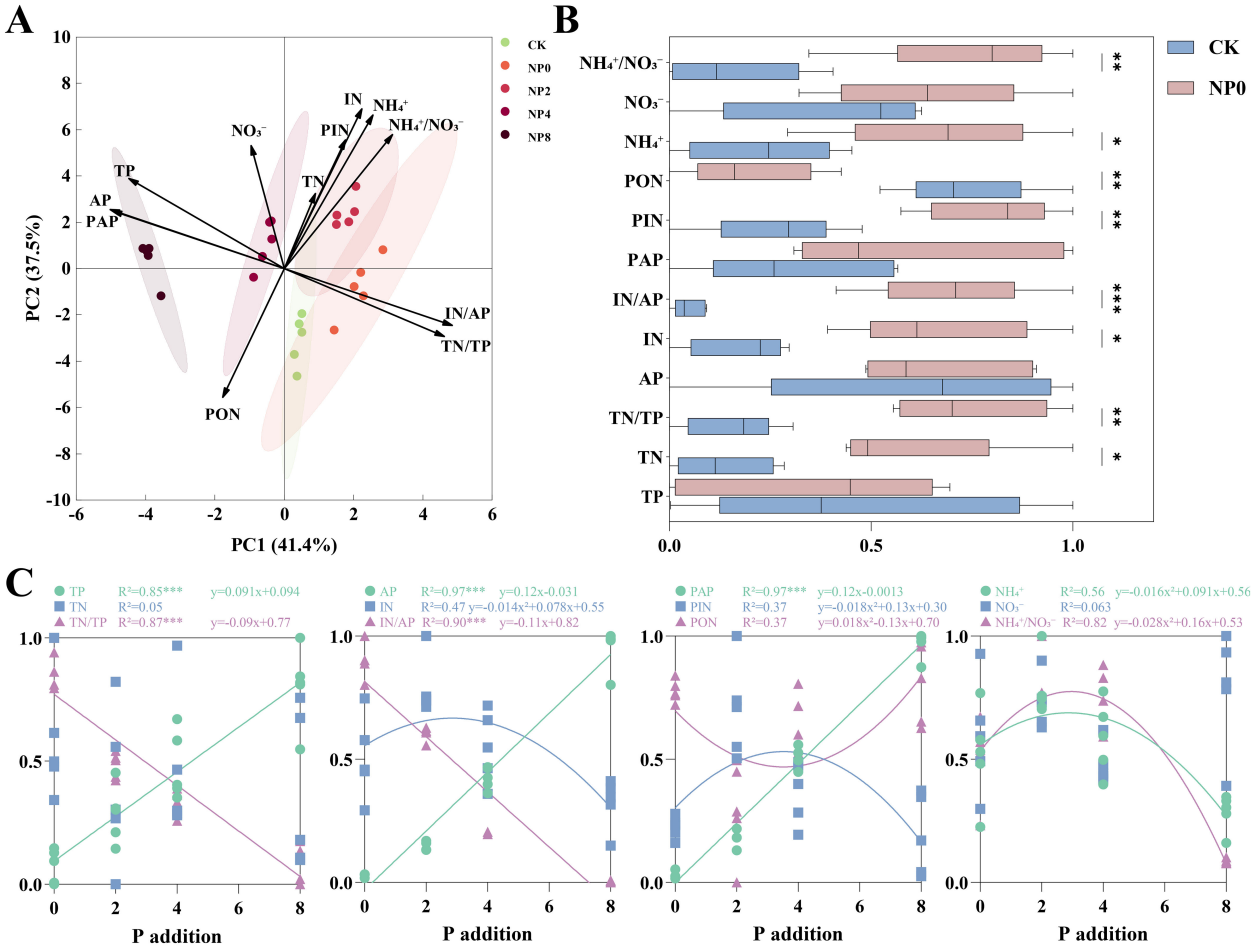
Response of soil physicochemical properties to NP addition. *P< 0.05, **P< 0.01, ***P< 0.001. TP, Total Phosphorus; TN, Total Nitrogen; AP, Available Phosphorus; IN, Inorganic Nitrogen; PAP, Proportion of Available Phosphorus; PIN, Proportion of Inorganic Nitrogen; PON, Proportion of Organic Nitrogen; NH_4_^+^, Ammonium Nitrogen; NO_3_^-^, Nitrate Nitrogen; NH_4_^+^/NO_3_^-^, Ammonium-Nitrate Ratio. Panel **(A)**: Principal Component Analysis (PCA) of soil samples. Panel **(B)**: Effect of N addition on soil samples. Panel **(C)**: Effect of NP addition on soil samples.

The PCA of soil physicochemical properties revealed that N addition (NP0, the same below) shifted soil physicochemical traits in the positive direction of N-related indicators. In contrast, NP addition (NP2, NP4, NP6, and NP8, the same below) shifted soil physicochemical traits along the P addition gradient in the positive direction of soil P-related indicators, with a greater shift distance than that under N addition alone (**Figure 1A**). Compared with CK, N addition significantly increased TN, TN/TP, IN, IN/AP, PIN, NH_4_^+^, and NH_4_^+^/NO_3_^-^, and significantly reduced IN/AP (**Figure 1B**). Under NP addition, soil TP, AP, and PAP increased linearly with the P addition gradient, whereas TN/TP and IN/AP decreased linearly. IN, PIN, NH_4_^+^, and NH_4_^+^/NO_3_^-^ first increased and then decreased along the P addition gradient, showing a single-peak curve, whereas PON first decreased and then increased. The fitted curves for the above indicators all peaked at a P addition rate between 2–4 g P·m^-2^ · a^-1^ (**Figure 1C**).

### 3.2 Differential responses of different plant functional traits to N and P Addition

**Figure 2:**
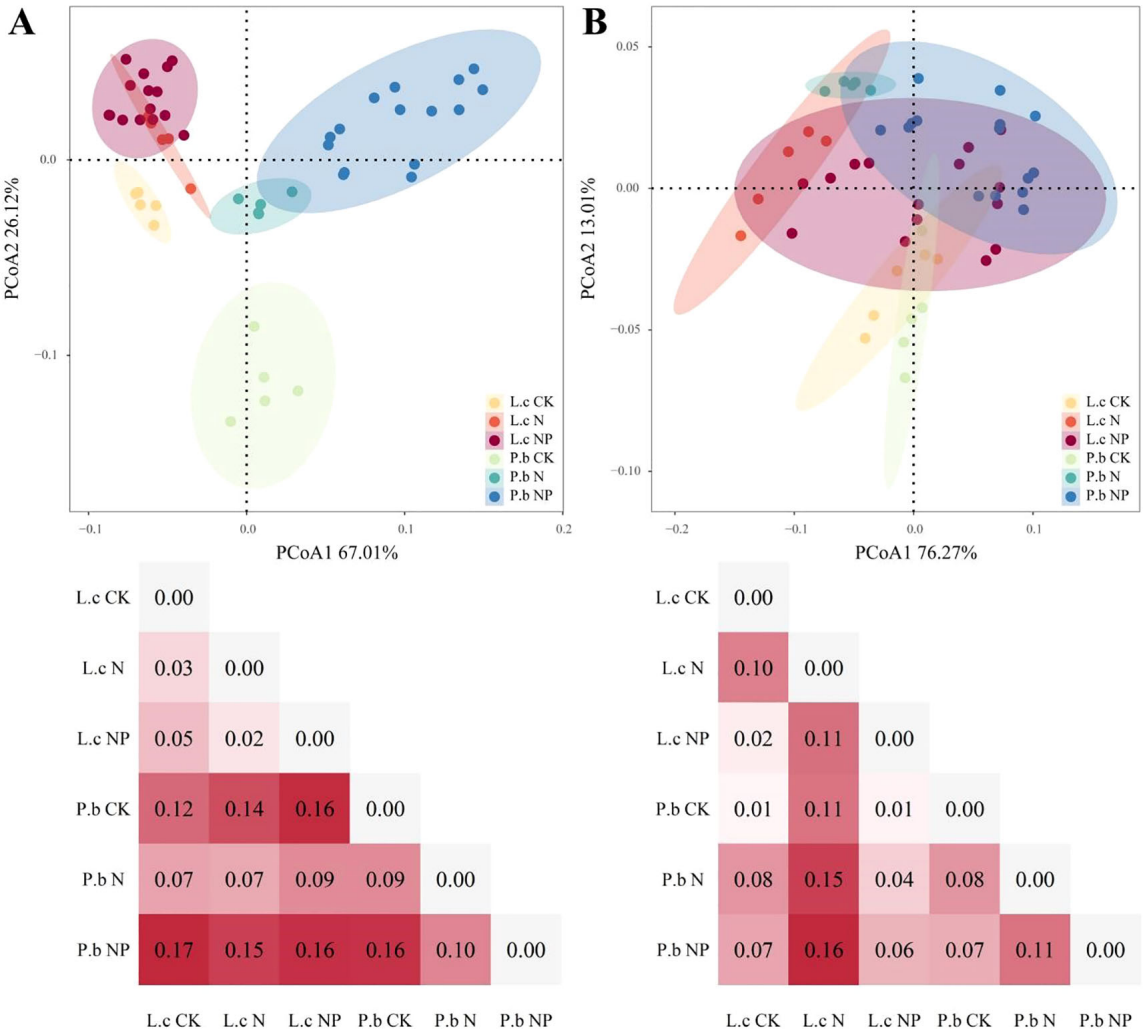
PCoA of plant phenotype and nutrient traits under different treatments. Panel **(A)**: The PCoA results of phenotypic traits. Panel **(B)**: The PCoA results of nutrient traits.

The PCoA results of phenotypic traits (**Figure 2A**) indicated that nutrient addition caused differential responses in the phenotypic traits of *L. chinensis* and *P. bifurca*. The phenotypic trait distances of *L. chinensis* between CK and N, N and NP, and CK and NP were 0.03, 0.02, and 0.05, respectively, while those of P. bifurca were 0.09, 0.10, and 0.16, respectively. These data reveal that the phenotypic trait differences of *P. bifurca* were greater than those of *L. chinensis*, reflecting its higher nutrient sensitivity. In addition, plant phenotypic traits can reflect the ecological niche relationships between plants to some extent. Under the N addition treatment, the 95% confidence intervals of phenotypic traits for *L. chinensis* and *P. bifurca* overlapped, and the trait distance decreased from 0.12 in CK to 0.07, indicating niche convergence between *L. chinensis* and *P. bifurca* under N addition. In contrast, under NP addition treatment, the 95% confidence intervals of phenotypic traits for *L. chinensis* and *P. bifurca* were separated, with the trait distance increasing from0.12 in CK to 0.16 in NP, indicating a trend of niche separation between *L. chinensis* and *P. bifurca* under NP addition (**Figure 2A**).

The PCoA results of nutrient traits (**Figure 2B**) showed that the nutrient trait distances of *L. chinensis* and *P. bifurca* were 0.10 and 0.08, respectively, between CK and N; 0.11 and 0.11 between N and NP; and 0.02 and 0.07 between CK and NP. Both species showed sensitivity to N addition (*L. chinensis*, 0.10; *P. bifurca*, 0.08); However, *P. bifurca* was more sensitive to NP addition (*P. bifurca*, 0.07 >*L. chinensis*, 0.02) (**Figure 2B**).

### 3.3 Plant stem–leaf nutrient allocation strategies showed differential responses to N and P addition

The CCorA results of soil nutrients and the related indicators of plant stem–leaf C, N, and P nutrient allocation strategies (**Figures 4A, B**) showed that the change directions of stem–leaf nutrient allocation in *L. chinensis* and *P. bifurca* were generally similar. Specifically, under N addition, the changes in stem–leaf nutrient allocation shifted toward the positive direction of soil N-related physicochemical indicators. Conversely, under NP addition, the changes in each stem–leaf trait indicator shifted toward the positive direction of soil P-related physicochemical indicators and continued to do so along the P addition gradient (except for *L. chinensis* under NP8 treatment).

The correlation analysis between soil nutrients and plant stem–leaf C, N, and P nutrient indicators (**Figures 4C, D**) also presented results similar to those of CCorA. The stem–leaf nutrient allocation indicators of *L. chinensis* were mainly positively correlated with soil N-related indicators, while those of *P. bifurca* were significantly correlated with both soil N- and P-related indicators. The stem–leaf P content ratio (SLTPCR) of *L. chinensis* was significantly negatively correlated with TP and AP, but significantly positively correlated with TN/TP and IN/AP. However, the SLTPCR of *P. bifurca* showed no significant correlation with soil nutrient traits.

The specific leaf area–biomass synergy index (SLABI) of *P. bifurca* was significantly positively correlated with TP and AP, and significantly negatively correlated with TN/TP and IN/AP, while that of *L. chinensis* was significantly positively correlated only with IN (**Figures 4C, D**).

### 3.4 Correlation analysis among functional traits of *L. chinensis* and *P. bifurca*

The results of Spearman correlation analysis showed that under nutrient addition, the correlations among the traits of *L. chinensis* and *P. bifurca* showed significant differences, with the correlation matrix of *P. bifurca* exhibiting a higher degree of data concentration (**Figures 5A, B**). Based on the numerical matrix of correlation coefficients (*R* values), further within-group correlation analysis models of all functional traits of the two species were established (**Figures 5C, D**). The results showed that, compared with *P. bifurca*, *L. chinensis* exhibited a smaller maximum sum of correlation coefficients across traits (43.5 for *L. chinensis*;<45.5 for *P. bifurca*) and a larger minimum sum (27.1 for *L. chinensis;* > 12.1 for *P. bifurca*), indicating that *L. chinensis* tends to have broader correlations among more functional traits. The histogram of absolute correlation coefficients (|*R*|) for the two species (**Figure 5E**) showed that *P. bifurca* exhibited peaks near both 0 and 1, i.e., at both low and high correlation ends. Contrastingly, *L. chinensis* exhibited a relatively uniform distribution of absolute *R* values across the 0–1 range, further indicating that *P. bifurca* has relatively strong correlations between certain individual functional traits.

Spearman correlation analysis using AGB as the core plant growth indicator showed that *L. chinensis* had 10 N-related and 2 P-related trait indices significantly correlated with AGB (**Figure 5A**), while *P. bifurca* had 3 N-related and 10 P-related trait indices significantly correlated with AGB (**Figure 5B**). This result indicates that the aboveground biomass (AGB) of *L. chinensis* is primarily governed by N-related traits, whereas that of *P. bifurca* is more dependent on P-related traits. This distinction is reinforced by the Random Forest models (**Figure 6**), which identified a broader array of traits (24 vs. >10) significantly associated with the AGB of *P. bifurca* (**Figures 6A, B**), with a notable emphasis on nutrient use efficiency (6 vs. >1 traits). The need to coordinate a more complex set of traits, particularly those governing nutrient efficiency, underscores a strategy of fine-tuned physiological regulation that may provide *P. bifurca* with a competitive advantage in nutrient-heterogeneous environments.

## 4 Discussion

### 4.1 N and P addition significantly altered the soil environment

P addition on the basis of N addition had a stronger effect on reshaping the soil nutrient environment than did N addition alone, revealing the important role of N–P interactions in nutrient cycling(**Figure 1A**). In the present study, the P addition gradient better reflected the N–P interaction effect (**Figure 1C**), with TP, AP, and PAP increasing linearly with P addition, indicating that P input improved soil P availability and relieved N–P imbalance (TN/TP significantly decreased). At the same time, N-related indicators such as IN, PIN, and NH_4_^+^ showed a non-linear “initial increase, then decrease” pattern, with peak values occurring at 2–4 g P·m^-2^ ·a^-1^, suggesting that N bioavailability was optimal at this P level. Higher P input or accumulation could reduce soil N availability. Mechanistically, at low P levels, P limitation was relieved and microbial metabolism was activated (e.g., increased phosphatase secretion), promoting organic N mineralization (increased IN and PIN) and ammonium production (Sun et al., 2014). At high P levels (NP8), excessive P reduced the soil TN/TP ratio, leading plants and microorganisms to absorb more N from the soil, thereby decreasing the residual inorganic N content (Marklein and Houlton, 2012; Dou et al., 2023). In addition, the “initial decrease, then increase” trend of PON supported this finding: under low P, microorganisms accelerated organic N decomposition to obtain P (PON decreased). Meanwhile, under high P, N became the limiting factor, prompting microorganisms to shift toward N retention (PON increased) (Mooshammer et al., 2014). These results suggest that a P input level of 2–4 g P·m^-2^ ·a^-1^ may be the threshold for maintaining soil N bioavailability in this region.

### 4.2 Interspecies differences in plant phenotypic plasticity and nutrient allocation strategies drive niche differentiation

**Figure 3:**
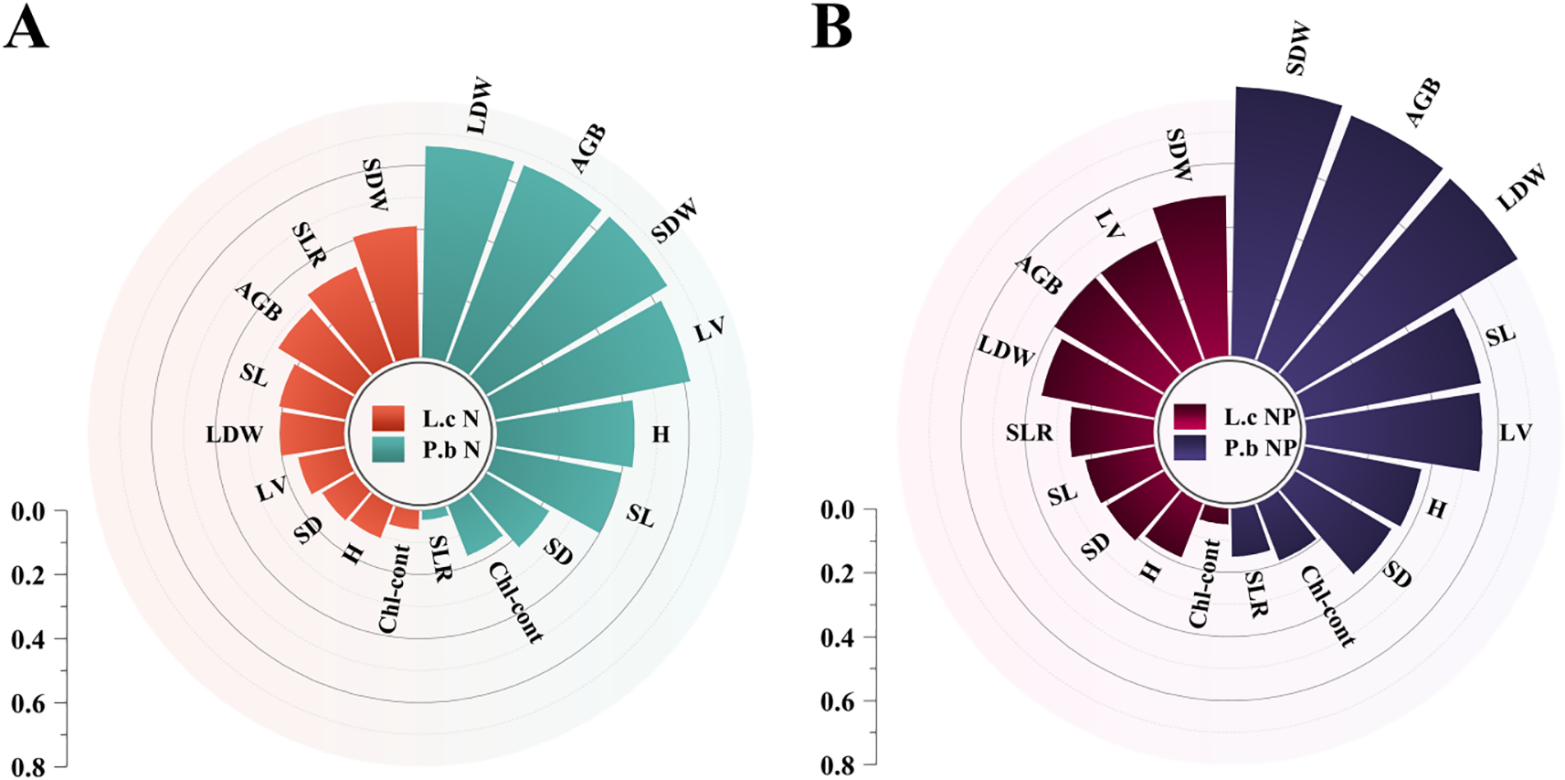
CCorA and correlation analysis of soil nutrients and plant stem and leaf traits. SLTCMR, Stem-to-Leaf Total Carbon Mass Ratio; SLTNMR, Stem-to-Leaf Total Nitrogen Mass Ratio; SLTPMR, Stem-to-Leaf Total Phosphorus Mass Ratio; SLTCCR, Stem-to-Leaf Total Carbon Content Ratio; SLTNCR, Stem-to-Leaf Total Nitrogen Content Ratio; SLTPCR, Stem-to-Leaf Total Phosphorus Content Ratio; LBER, Leaf Biomass Efficiency; PLA, Productivity per Leaf Area; SLABI, Specific Leaf Area–Biomass Index; PSE, Photosynthetic-Structural Efficiency. Panel **(A)**: Plant plasticity under N addition. Panel **(B)**: Plant plasticity under NP addition.

Under N and P addition, *L. chinensis* and *P. bifurca* exhibited significant interspecies differences in both phenotypic responses and nutrient allocation strategies, which profoundly influenced their resource use efficiency and interspecific competition. *P. bifurca* consistently displayed much higher phenotypic plasticity than that of *L. chinensis*, especially in key growth traits such as AGB, LDW, and SDW (**Figures 2B**, **3**, **6**). This higher plasticity enables *P. bifurca* to rapidly adjust morphological traits to capture resources (e.g., light and nutrients), reflecting a proactive adaptive strategy of a subdominant understory species in the face of environmental disturbance (Valladares et al., 2007; Nicotra et al., 2010). In contrast, *L. chinensis*, as a dominant overstory species, maintains a stable phenotypic strategy that helps secure access to upper-layer resources (Grassein et al., 2010; Paez-Garcia et al., 2015). N addition reduced the differences in phenotypic traits between the two species (niche distance decreased from 0.12 to 0.07), with the 95% confidence intervals overlapping (**Figure 2A**), indicating niche convergence and potentially intensifying interspecific competition. However, combined N and P addition altered this competitive pattern: through stronger phenotypic adjustments (e.g., increased AGB plasticity index), *P. bifurca* achieved significant trait-space separation from *L. chinensis* (trait distance expanded to 0.16), thereby relieving interspecific competition. This phenomenon is consistent with the “resource partitioning hypothesis,” (Lefcheck et al., 2015; Vallicrosa et al., 2023) whereby, under the abundant NP resources in the present study, the two species exhibited niche separation, reducing direct competition. The greater plasticity of multiple traits in *P. bifurca* emerged as a key strategy for mitigating competition with *L. chinensis*.

**Figure 4:**
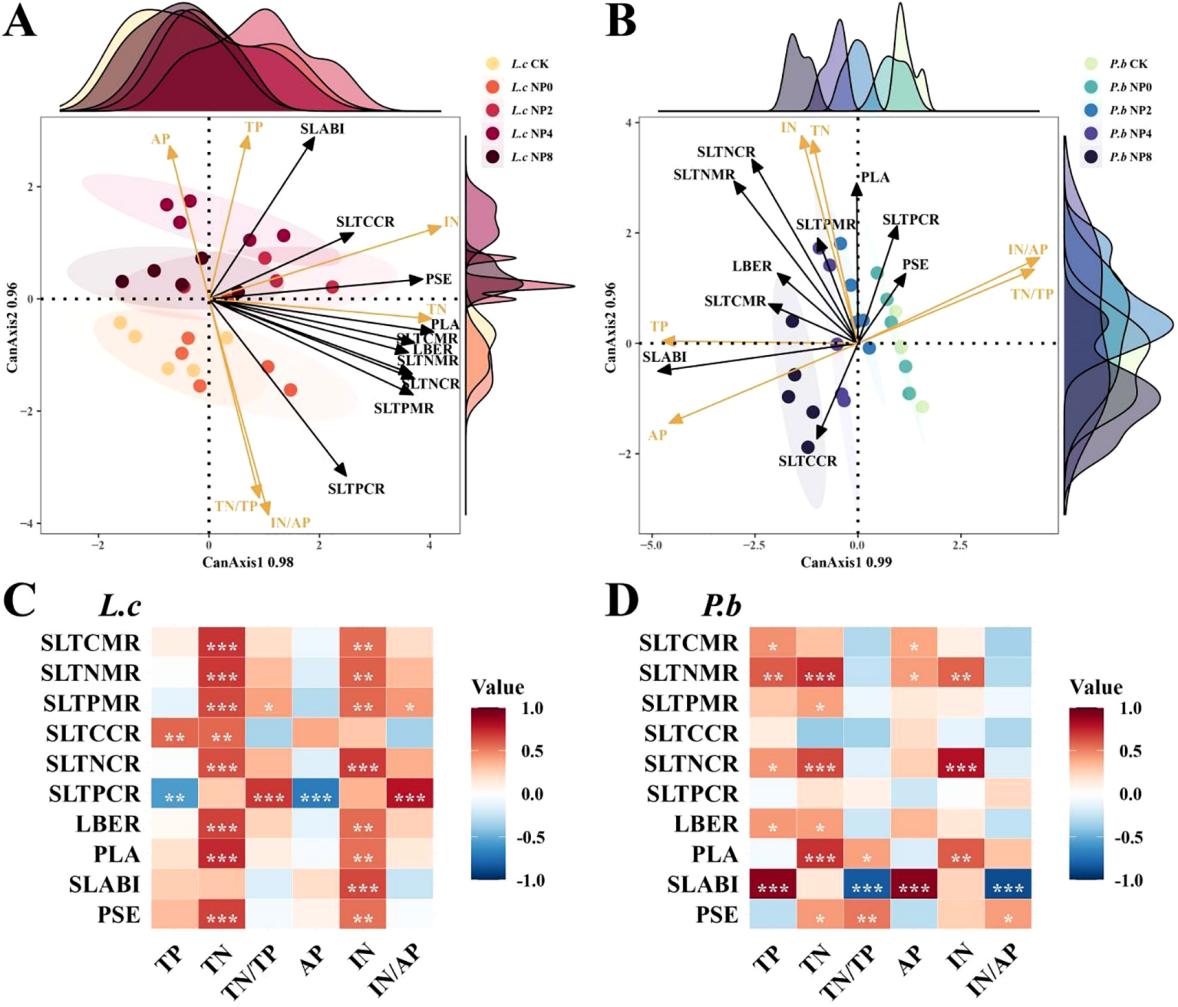
Correlation analysis between soil nutrients. *P< 0.05, **P< 0.01, ***P< 0.001. PCC, Plant Carbon Content; PNC, Plant Nitrogen Content; PPC, Plant Phosphorus Content; SCC, Stem Carbon Content; SNC, Stem Nitrogen Content; SPC, Stem Phosphorus Content; LCC, Leaf Carbon Content; LNC, Leaf Nitrogen Content; LPC, Leaf Phosphorus Content; PN/P, Plant N:P Ratio; PC/N, Plant C:N Ratio; PC/P, Plant C:P Ratio; TPCM, Total Plant Carbon Mass; TPNM, Total Plant Nitrogen Mass; TPPM, Total Plant Phosphorus Mass; TSCM, Total Stem Carbon Mass; TSNM, Total Stem Nitrogen Mass; TSPM, Total Stem Phosphorus Mass; TLCM, Total Leaf Carbon Mass; TLNM, Total Leaf Nitrogen Mass; TLPM, Total Leaf Phosphorus Mass; PNNER, Plant N Nutrient Efficiency Ratio; PPNER, Plant P Nutrient Efficiency Ratio; LNNER, Leaf N Nutrient Efficiency Ratio; LPNER, Leaf P Nutrient Efficiency Ratio; SNNER, Stem N Nutrient Efficiency Ratio; SPNER, Stem P Nutrient Efficiency Ratio. Panel **(A)**: Canonical correspondence analysis (CCorA) ordination plot of L. c and soil. Panel **(B)**: Canonical correspondence analysis (CCorA) ordination plot of P. b and soil. Panel **(C)**: Correlation heatmap of L. c and soil. Panel **(D)**: Correlation heatmap of P. b and soil.

Although the stem–leaf nutrient allocation of the two species responds similarly to changes in soil nutrients (**Figures 4A, B**), their specific mechanisms exhibit essential differences. Our results uncover a fundamental mechanistic link between soil N/P stoichiometry and plant niche differentiation. Nitrogen addition intensified soil P limitation (high N/P ratio), which constrained the P-sensitive *P. bifurca*. The resulting niche convergence suggests intensified competition for limited P resources. In contrast, combined NP addition, particularly at moderate levels (2–4 g P m^-2^ a^-1^), optimized soil nutrient conditions by alleviating P limitation and enhancing N bioavailability, creating an opportunity for niche partitioning. The key to this shift lies in the divergent adaptive strategies of the two species. *P. bifurca* exhibited a continuous, positive response to P addition, as evidenced by the strong correlation between its specific leaf area–biomass synergy index (SLABI) and soil available P. This suggests that *P. bifurca* possesses a superior ability to acquire and utilize supplemental P, potentially through mechanisms such as root morphology adjustments or associations with phosphorus-solubilizing microbes, a strategy crucial for an understory species competing for belowground resources (Han et al., 2022). Conversely, *L. chinensis*, as the canopy-dominant species, maintained a stable growth strategy reliant on N availability. Its negative response to high P levels (NP8), indicated by the significant negative correlation between stem-leaf P content ratio (SLTPCR) and soil P, may be attributed to ion antagonism (e.g., P-Zn interactions) that disrupts internal nutrient balance (Vance et al., 2003; Moda et al., 2021). Thus, the higher phenotypic plasticity and efficient P-use strategy of *P. bifurca* under balanced NP supply enabled it to exploit a distinct resource axis, thereby facilitating niche separation and reducing direct competition with the N-focused *L. chinensis*.

### 4.3 Response mechanisms of soil nutrient changes and plant adaptive strategies

**Figure 5:**
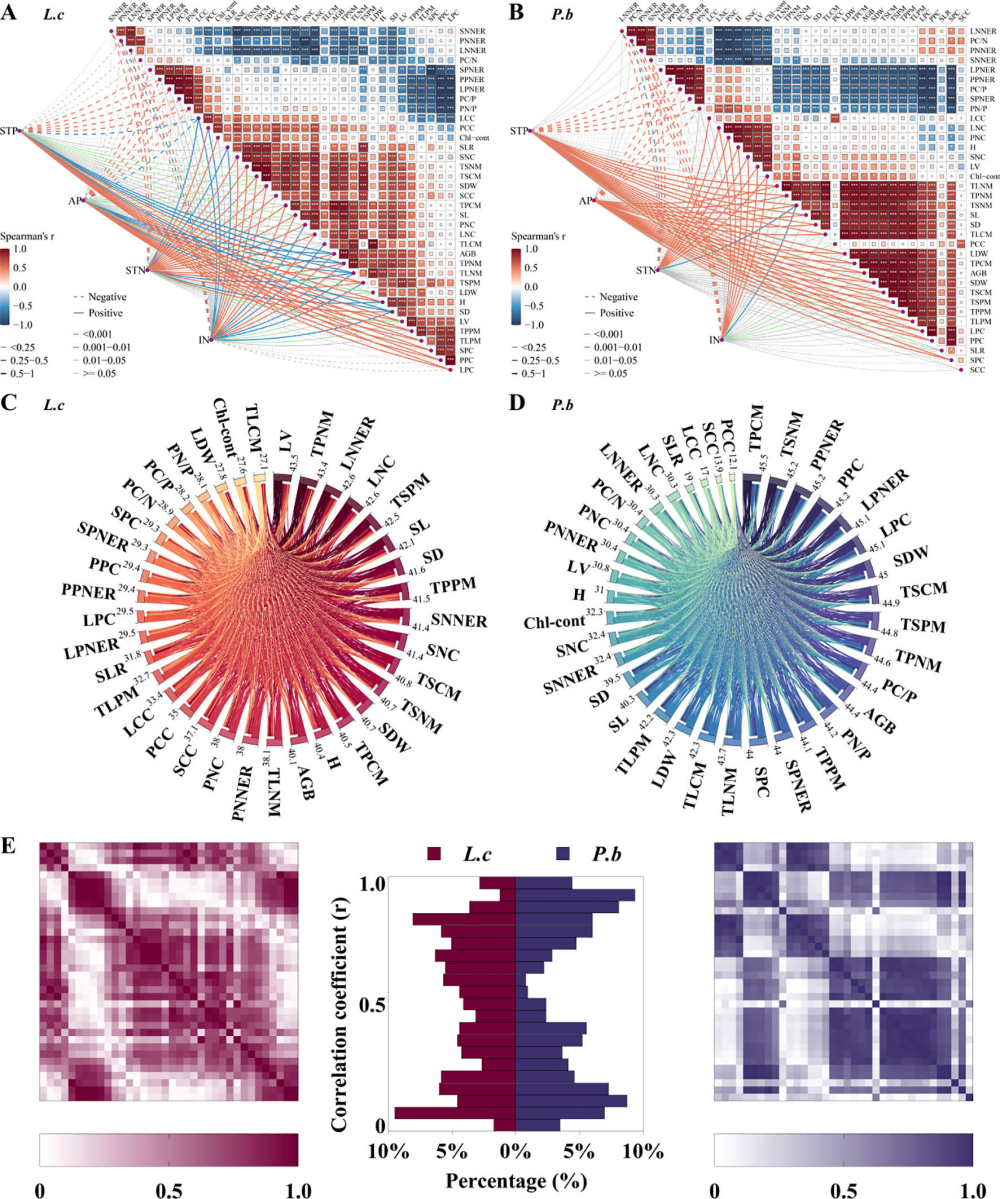
Plant functional traits and random forest model of aboveground biomass. Panel **(A)**: Spearman correlation heatmap of L.c. Panel **(B)**: Spearman correlation heatmap of P.b. Panel **(C)**: All correlation matrix analysis models of L.c. Panel **(D)**: All correlation matrix analysis models of P.b. Panel **(E)**: Frequency distribution histogram of the correlation matrices of L.c and P.b.

N and P additions significantly alter soil N and P availability and their ratio (N/P), creating N- or P-limited environments in the soil. *L. chinensis* and *P. bifurca*, as the upper dominant and lower subdominant species in the community, respectively, have distinct environmental adaptation strategies and response mechanisms. N addition intensifies P limitation by increasing soil TN, IN, and TN/TP (**Figure 1B**). Owning to the widespread and relatively balanced correlations among its functional traits (**Figure 5C**: correlation coefficients were evenly distributed with a small range: 43.5–27.1 = 16.4) and its AGB regulation mechanism centered on N use efficiency (**Figure 5A**: 10 N-related traits, 2 P-related traits), *L. chinensis* maintains relatively high resource acquisition and growth under conditions of relative N enrichment and P limitation. In contrast, due to its strong P response characteristics (**Figure 5B**: AGB driven by 10 P-related traits) and a stem N nutrient efficiency ratio lower than that of *L. chinensis*, *P. bifurca* shows inhibited growth under P limitation. Under the circumstance, no significant niche differentiation occurs between the two species, and interspecific competition intensifies.

**Figure 6:**
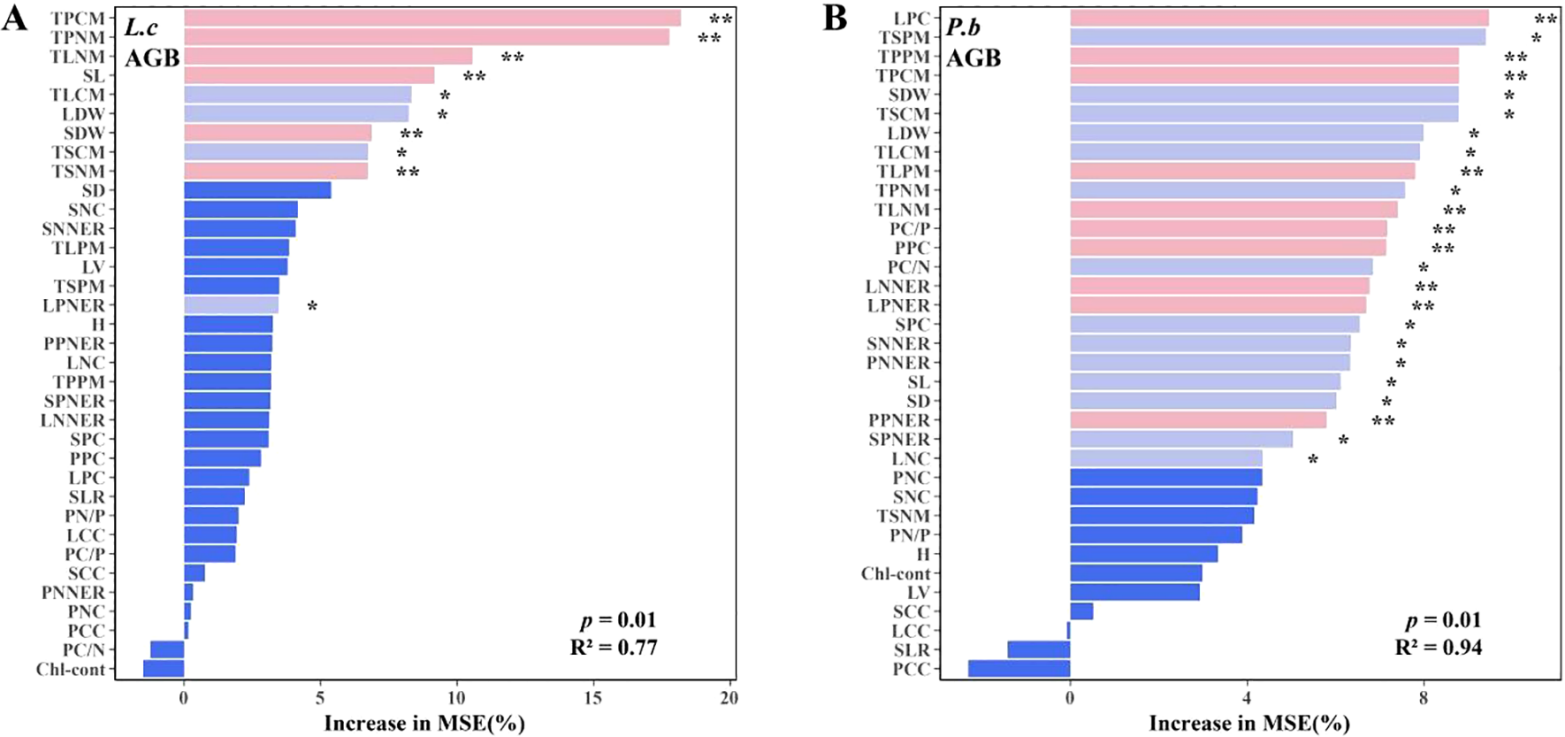
Plant phenotypic plasticity index under N and P addition. **(A)** Plasticity indices of plant functional traits under N addition. **(B)** Plasticity indices of plant functional traits under combined N and P addition. AGB, Aboveground Biomass per Individual; H, Plant Height; SDW, Stem Dry Weight; LDW, Leaf Dry Weight; SL, Stem Length; SD, Stem Diameter; LV, Leaf Volume; SLR, Stem-to-Leaf Ratio; Chl-cont, Chlorophyll Content. Panel **(A)**: Random Forest models of L.c's AGB. Panel **(B)**: Random Forest models of P.b's AGB.

Low P level treatments (2–4 g P m^-2^·a^-1^) linearly increase soil TP and AP and significantly decrease the N/P ratio, while soil IN peaks. Because *P. bifurca* AGB is driven by more P-related indicators (**Figure 5B**: driven by 10 P traits) and has higher nutrient use efficiency characteristics (**Figure 6**), its key plasticity indicators, such as AGB, SDW, LDW, and SL, increase. Meanwhile, N nutrient efficiency ratio indicators such as PNNER, LNNER, and SNNER in *P. bifurca* increased significantly. These advantages drive a significant separation in phenotypic space between *P. bifurca* and *L. chinensis*, achieving niche differentiation and effectively relieving competition pressure. High P input (NP8) may cause intensified N limitation (N/P lower than CK) or side effects such as ion antagonism (Zeng et al., 2021). At this stage, the SLTPCR of *L. chinensis* is significantly negatively correlated with soil TP and AP (**Figure 4C**), indicating that high P may inhibit its effective P absorption or utilization. This may be due to ion antagonism caused by high P levels, leading to Zn deficiency in the plant (Sun et al., 2013) or affecting N metabolism (**Figure 4A**: NP8 treatment response direction deviates) (Güsewell, 2004). P*. bifurca* benefits from its sensitive P response ability and regulation mechanism involving multiple traits (**Figure 6B**: 24 traits regulating AGB), giving it higher environmental adaptability.

## 5 Conclusion

In conclusion, our study demonstrates that long-term N and P additions regulate interspecific interactions in a temperate meadow steppe through a cascade of effects: by altering soil N/P stoichiometry, they induce species-specific shifts in phenotypic plasticity and nutrient allocation, ultimately driving niche convergence or separation. We showed that the subdominant *P. bifurca*, leveraging its higher plasticity and more efficient P-use strategy, capitalizes on combined NP addition to achieve niche differentiation from the dominant *L. chinensis*. Thus, while focused on two key species, our study provides a mechanistic understanding of how nutrient stoichiometry can rewire species interactions. Future research integrating a broader range of species, belowground traits, and rhizosphere processes will be essential to build a comprehensive predictive model of community reassembly under global nutrient enrichment.

## Data Availability

The original contributions presented in the study are included in the article/supplementary material. Further inquiries can be directed to the corresponding author.
